# Supplementary figures and images for: Identification of miRNAs Involved in Maize-Induced Systemic Resistance Primed by Trichoderma harzianum T28 against Cochliobolus heterostrophus
Source: J Fungi (Basel). 2023 Feb 20;9(2):278. doi: 10.3390/jof9020278 (PMC9964586; doi:10.3390/jof9020278)

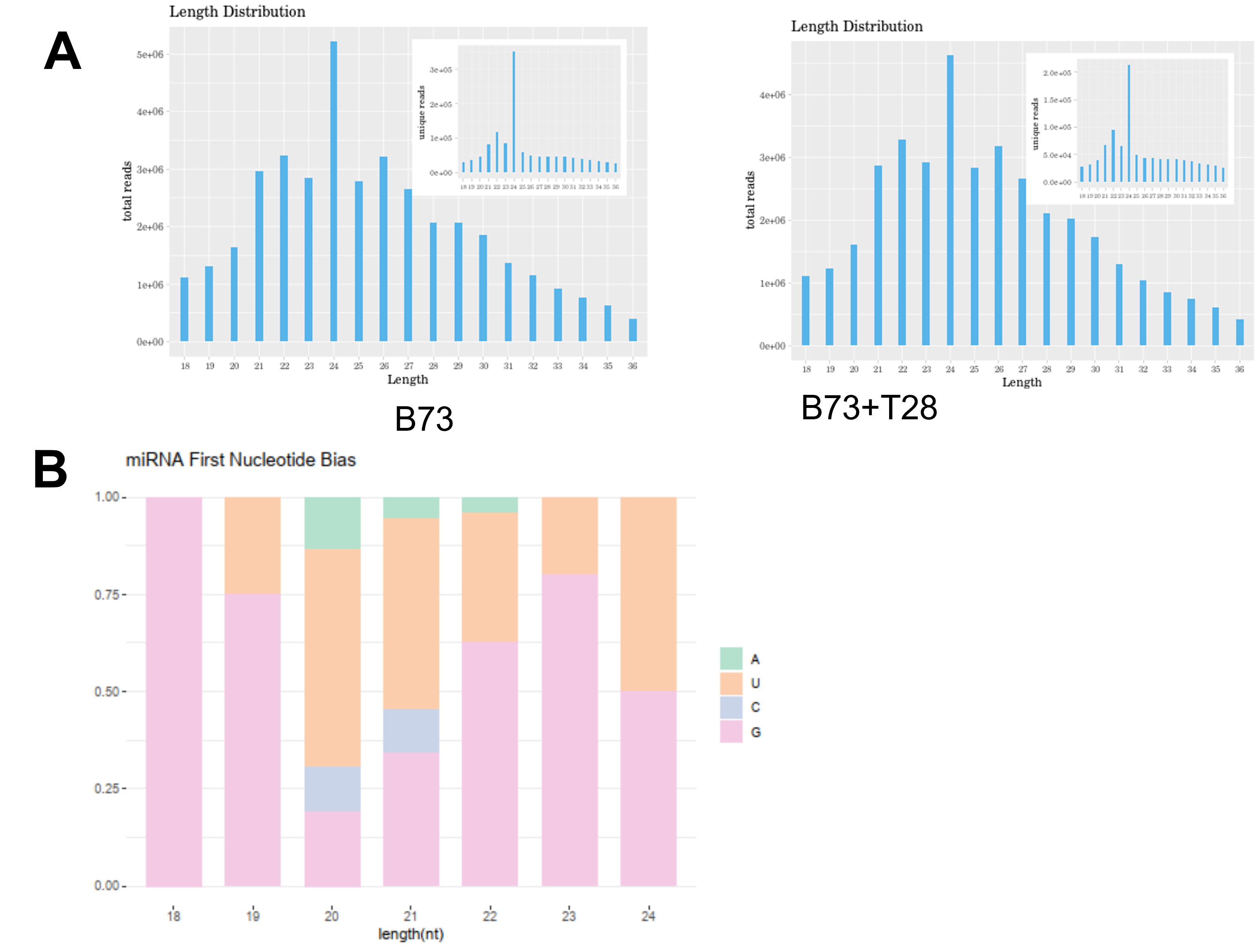

Supplement: Supplementary file 1 [file jof-09-00278-s001.zip › Figure s1.tif]
